# Meta-Analysis of Mid-*p*-Values: Some New Results based on the Convex Order

**DOI:** 10.1080/01621459.2018.1469994

**Published:** 2018-08-06

**Authors:** Patrick Rubin-Delanchy, Nicholas A. Heard, Daniel J. Lawson

**Affiliations:** a School of Mathematics, University of Bristol, Heilbronn Institute for Mathematical Research, Bristol, United Kingdom; b Department of Mathematics, Imperial College London, Heilbronn Institute for Mathematical Research, London, United Kingdom; c Integrative Epidemiology Unit, Population Health Sciences, University of Bristol, Bristol, United Kingdom

**Keywords:** Convex order, Cyber-security, Meta-analysis, Network, *p*-Value

## Abstract

The mid-*p*-value is a proposed improvement on the ordinary *p*-value for the case where the test statistic is partially or completely discrete. In this case, the ordinary *p*-value is conservative, meaning that its null distribution is larger than a uniform distribution on the unit interval, in the usual stochastic order. The mid-*p*-value is not conservative. However, its null distribution is dominated by the uniform distribution in a different stochastic order, called the convex order. The property leads us to discover some new finite-sample and asymptotic bounds on functions of mid-*p*-values, which can be used to combine results from different hypothesis tests conservatively, yet more powerfully, using mid-*p*-values rather than *p*-values. Our methodology is demonstrated on real data from a cyber-security application.

## 1. Introduction

Let *T* be a real-valued test statistic, with probability measure P_0_ under the null hypothesis, denoted *H*_0_. Let *X* be a uniform random variable on the unit interval that is independent of *T* under P_0_. *X* is a randomization device which is in practice usually generated by a computer.

We consider the (one-sided) *p*-value,

(1) $$P=P_{0}\left(T^{*}\geq T\right),$$

the mid-*p*-value (Lancaster 1952),

(2) $$Q=\frac{1}{2}P_{0}\left(T^{*}\geq T\right)+\frac{1}{2}P_{0}\left(T^{*}>T\right),$$

and the randomized *p*-value,

(3) $$R=XP_{0}\left(T^{*}\geq T\right)+\left(1-X\right)P_{0}\left(T^{*}>T\right),$$

where T* is a hypothetical independent replicate of *T* under P_0_. If *T* is absolutely continuous under *H*_0_, then the three quantities are equal and distributed uniformly on the unit interval. More generally, that is, if discrete components are possible, the three are different. Two main factors, one obvious and one more subtle, make this a very common occurrence. First, *T* is discrete if it is a function of discrete data, for example, a contingency table, categorical data, or a presence/absence event. Second, discrete test statistics often occur as a result of conditioning, as in the permutation test or Kendall’s tau test (Sheskin 2003). Partially discrete tests occur, for example, as a result of censoring.

When *P*, *Q*, and *R* are not equal, it is a question which to choose. The ordinary *p*-value is often preferred in relatively strict hypothesis testing conditions, for example, in clinical trials, where the probability of rejecting the null hypothesis must not exceed the nominal level (often 5%). The randomized *p*-value has some theoretical advantages, for example, the nominal level of the test is met exactly. However, to quote one of its earliest proponents, “most people will find repugnant the idea of adding yet another random element to a result which is already subject to the errors of random sampling” (Stevens 1950). Randomized *p*-values also fail Birnbaum’s admissibility criterion (Birnbaum 1954). Note that we can also work with an unrealized version of the randomized *p*-value, known as the *fuzzy* or *abstract* *p*-value (Geyer and Meeden 2005), and either stop there—leaving interpretation to the decision-maker—or propagate uncertainty through to any subsequent analysis, for example, multiple-testing (Kulinskaya and Lewin 2009; Habiger 2015).

Although it can allow breaches of the nominal level, the mid-*p*-value is often deemed to better represent evidence against the null hypothesis than the ordinary or randomized *p*-values. Justifications are not just heuristic as, for example, the mid-*p*-value can arise as a Rao–Blackwellization of the randomized *p*-value corresponding to the uniformly most powerful test (Wells 2010), as an optimal estimate of the *H*_0_ versus *H*_1_ truth indicator under squared loss (Hwang and Yang 2001), or from asymptotic Bayesian arguments (Routledge 1994). Performance has also been demonstrated in applications, for example, in the context of healthcare monitoring (Spiegelhalter et al. 2012) (an article read before the Royal Statistical Society), genetics (Graffelman and Moreno 2013), a wealth of examples involving contingency tables (Lydersen, Fagerland, and Laake 2009), and more. Our own interest stems from cyber-security applications, and a motivating example is given in Section 3. Most arguments for using the mid-*p*-value in hypothesis testing scenarios also work for confidence intervals. Here, using the mid-*p*-value over the *p*-value can result in a smaller interval, with a closer-to-nominal coverage probability (Berry and Armitage 1995; Fagerland, Lydersen, and Laake 2015).

In this article, we are able to make further mathematical progress on the mid-*p*-value by using a stochastic order known as the *convex order*. The problem we focus on is meta-analysis, that is, combining evidence from different hypothesis tests into one, global measure of significance. In several scenarios analyzed, the use of the ordinary *p*-value leads to suboptimal, and even spurious results. New bounds for some commonly used methods for combining ordinary *p*-values are derived for mid-*p*-values. These allow large gains in power over using ordinary *p*-values, while, unlike any previous study based on mid-*p*-values, the false positive rate is still controlled exactly (albeit conservatively).

The remainder of this article is structured as follows. In Section 2, we summarize our main results. Section 3 gives a cyber-security application where, using mid-*p*-values, we are able to detect a cyber-attack that would likely fall under the radar if only ordinary *p*-values were used. Section 4 elaborates on the results of Section 3, with improved (although more complicated) bounds, simulations, and discussion. Section 5 concludes. All proofs are relegated to the Appendix.

## 2. Main Results

This section summarizes the main ideas and findings of the article. Let *U* denote a uniform random variable on the unit interval, with expectation operator E, and let E_0_ denote expectation with respect to P_0_. Under the null hypothesis, it is well known, see, for example, Casella and Berger (2002), that *P* dominates *U* in the *usual stochastic order*, denoted *P* ⩾ _*st*_*U*. One way to write this is

(4) $$E_{0}\left\{f\left(P\right)\right\}\geq E\left\{f\left(U\right)\right\},$$

for any nondecreasing function *f*, whenever the expectations exist (Shaked and Shanthikumar 2007). It is also well known, and in fact true by design, that *R* is uniformly distributed under the null hypothesis, denoted *R* = _*st*_*U*. On the other hand, it is not widely known that, under the null hypothesis, *Q* is dominated by *U* in the *convex order*, denoted *Q* ⩽ _*cx*_*U*. One way to write this is (Shaked and Shanthikumar 2007, chap. 3)

(5) $$E_{0}\left\{h\left(Q\right)\right\}\leq E\left\{h\left(U\right)\right\},$$

for any *convex* function *h*, whenever the expectations exist. We have used the qualifier “widely,” because an effective equivalent of Equation (5) can be found in Hwang and Yang (2001). However, even there, Equation (5) is not recognized as a major stochastic order, meaning that some of its importance is missed.

In particular, we now present three concrete, new results, made possible by the literature on the convex order. Each provides a method for combining mid-*p*-values conservatively, the first two in finite samples and the last asymptotically. Details and improved (but more complicated) bounds are given in Section 4. In what follows, *Q*_1_, …, *Q_n_* denote independent (but not necessarily identically distributed) mid-*p*-values, with joint probability measure denoted ${\tilde{P}}_{0}$ under the null hypothesis.

Let ${\bar{Q}}_{n}=n^{-1}\sum_{i=1}^{n}Q_{i}$ denote the average mid-*p*-value. For *t* ⩾ 0,

(6) $${\tilde{P}}_{0}\left(1/2-{\bar{Q}}_{n}\geq t\right)\leq \exp\left(-6nt^{2}\right).$$

Note that, first, no knowledge of the specific individual mid-*p*-value distributions is required. Second, Hoeffding’s inequality (Hoeffding 1963), which would be available more generally, gives the larger bound exp ( − 2*nt*^2^) (the cubic root).

Let *F_n_* = −2∑^*n*^_*i* = 1_log (*Q_i_*), known as Fisher’s statistic (Fisher 1934) and the most popular method for combining *p*-values. In the continuous case, it is well-known that *F_n_* has a chi-square distribution with 2*n* degrees of freedom under *H*_0_. For *t* ⩾ 2*n*,

(7) $${\tilde{P}}_{0}\left(F_{n}\geq t\right)\leq \exp\left\{n-t/2-n\log\left(2n/t\right)\right\}.$$

Finally, assume additionally that *Q*_1_, …, *Q_n_* are identically distributed. Then applying Fisher’s method as usual, that is, treating the mid-*p*-values as if they were ordinary *p*-values and using the chi-square tail, is asymptotically conservative as *n* → ∞.

## 3. Example: Network Intrusion Detection

The perceived importance of cyber-security research has risen dramatically in recent years, particularly after several well-publicized events in 2016 and 2017. In this area, anomaly detection over very high volumes and rates of network data is a key statistical problem (Adams and Heard 2016). In our experience of the field, discrete data, whether they be presence/absence events, counts or categorical data, are the norm rather than the exception. We will demonstrate the value of our article’s contributions in a network intrusion detection problem.

**Figure 1.**
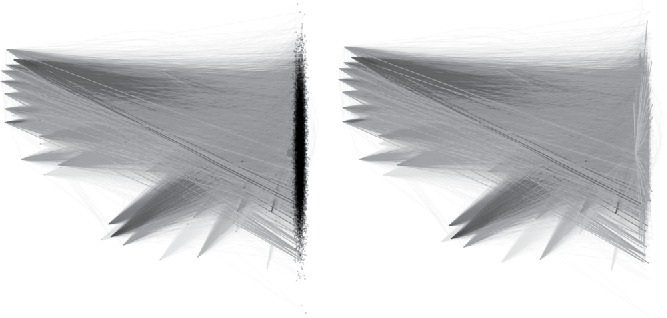
Authentication data: Full network of connections comprising ∼18,000 nodes and ∼400,000 directed edges. Edges are colored by authentication type. On the left, nodes are shown as black points, with node ID “C17693” highlighted in red (and larger). On the right, the points are hidden to better see the connections made by node ID “C17693,” which are now highlighted in pink.

Figure 1 shows publically available authentication data covering 58 days on the Los Alamos National Laboratory computer network (Kent 2016). Nodes in the graph are computers, and an edge indicates that there was at least one connection from one computer to the other, resulting in a graph with *m* ≈ 18,000 nodes and ∼400,000 directed edges. An exciting opportunity offered by this data resource is that it contains an actual cyber-attack: or, to be precise, records of penetration testing activity conducted by a “red-team.” One of the four computers used for the attack (the highest degree of the four, ID “C17693,” with 296 out of 534 edges labeled as nefarious) is highlighted in red on the left, with its connections highlighted in pink on the right.

Earlier work on network intrusion has suggested that the occurrence of *new edges* on the network can be (weakly) indicative of nefarious behavior (Neil et al. 2013; Neil 2015). Looking at the outward connections from a given computer, in particular, those which at the other end involve a computer otherwise receiving relatively few new connections present special interest. Because the first day of data has no red-team activity, we use this day to learn a rate λ_*j*_, *j* = 1, …, *m* at which each computer receives new connections, assuming, admittedly unrealistically, that the times are right-censored independent and identically distributed exponential random variables. For every computer on the network, the set of outward new connections made over the remainder of the observation period [1, 58] is scored according to this model. The test-statistic

$$T_{ij}=\left\{\begin{array}{cc} 57 & \text{if}\;\text{no}\;\text{connection}\;\text{occurs}\;\text{from}\;i\;\text{to}\;j\text{,}\\ \tau -1 & \text{if}\;\text{a}\;\text{new}\;\text{connection}\;\text{from}\;i\;\text{to}\;j\;\text{occurs}\\ & \;\text{at}\;\text{time}\;\tau \geq 1, \end{array}\right.$$

is considered for every directed pair (*i*, *j*) not occurring as an edge on the first day, so that each node *i* has associated with it a collection of test statistics *T*_*i* ·_, which are partially discrete, with a point mass at 57.

For regularization purposes, the rates λ_*j*_, *j* = 1, …, *m* are assumed a priori to follow a Gamma distribution matching the mean and variance of the empirical rates computed for each *j* = 1, …, *m* over the full period of 58 days. The use of this prior implies that before censoring *T_ij_* has a Gamma-Exponential (also called Lomax) predictive distribution, which is used to compute the collection of ordinary, mid, and randomized *p*-values *P*_*i* ·_, *Q*_*i* ·_, *R*_*i* ·_ corresponding to the outward connections of each node *i* = 1, …, *m*. Mathematical details about the calculations above are in the Appendix.

Since we are interested in the *ranking* of computer ID “C17693” among the other ∼18,000 computers, as well as its *p*-value, it makes sense to extend the ranges of the bounds (6) and (7) as follows:

(8) $$\;\;\;\;\;\;\;\;\;\;\;\;\;\;\;\;{\tilde{P}}_{0}\left(1/2-{\bar{Q}}_{n}\geq t\right)\leq \exp\left\{-6\mathrm{sgn}\left(t\right)nt^{2}\right\},\;\;t\in R,$$

(9) $$\;\;\;\;\;\;\;\;\;\;\;\;\;\;\;\;{\tilde{P}}_{0}\left(F_{n}\geq t\right)\leq \exp\left[\mathrm{sgn}\left(t-2n\right)\left\{n-t/2-n\log\left(2n/t\right)\right\}\right],\;t>0,$$

which preserves monotonicity, and remains valid because larger values than unity are returned outside the old ranges. Our options are:

1. to compute the average ordinary, mid, and randomized *p*-values, and obtain a significance level using bound (8). Computer ID “C17693” then ranks as 8th (*p*-value ≈ 1), 8th (*p*-value ≈ 10^− 7^), and 9th (*p*-value ≈ 10^− 7^) most anomalous of the ∼18,000 computers, respectively.
2. to compute Fisher’s statistic for the ordinary, mid, and randomized *p*-values, and obtain a global significance level using bound (9) for the second case, and the chi-square tail otherwise. Computer ID “C17693” now ranks joint 8118th (*p*-value ≈ 1), 2nd (*p*-value ≈ 1), and 9th (*p*-value ≈ 10^− 43^), respectively.
3. to assume an asymptotic regime and use the chi-square tail for the Fisher-with-mid-*p*-values statistic instead. Computer ID “C17693” then ranks 8th (*p*-value ≈ 1).

As rankings go, therefore, the mid-*p*-value is never beaten, with computer ID “C17693” coming in the top 10 every time and coming second once. The most obvious approach of using Fisher’s method with ordinary *p*-values fails completely. As for the other three red-team computers: using the best performing method, that is, Fisher’s statistic with mid-*p*-values and bound (9), where Computer ID “C17693” comes second, their ranks are 384th (ID “C18025”), 550th (ID “C19932”), and 1079th (ID “C22409”).

## 4. Meta-Analysis of Mid-*p*-Values: Further Details

This section elaborates on the results of Section 2. We say that a random variable (and its measure and distribution function) is *subuniform* if it is less variable than a uniform random variable, *U*, in the convex order.

To see why the mid-*p*-value is sub-uniform, notice that *Q* = E_0_(R∣T). By Jensen’s inequality, for any convex function *h*,

(10) $$\begin{array}{ccc} E_{0}\left\{h\left(Q\right)\right\} & = & E_{0}\left[h\left\{E_{0}\left(R|T\right)\right\}\right]\leq E_{0}\left[E_{0}\left\{h\left(R\right)|T\right\}\right]\\ & = & E_{0}\left\{h\left(R\right)\right\}=E\left\{h\left(U\right)\right\}, \end{array}$$

whenever the expectations exist, since *R* = _*st*_*U*. Remember that we do not claim this result is new, see, for example, Hwang and Yang (2001), but rather the idea to exploit the literature on the convex order.

To formalize the meta-analysis framework, let *T*_1_, …, *T_n_* be a sequence of independent test statistics. We consider a joint null hypothesis, ${\tilde{H}}_{0}$, under which *T*_1_, …, *T_n_* have probability measure $P_{0}^{\left(1\right)},\ldots ,P_{0}^{\left(n\right)}$, respectively. The *p*-values, *P_i_*, mid-*p*-values, *Q_i_*, and randomized *p*-values, *R_i_*, are obtained by replacing P_0_ with P^(i)^_0_ in (1), (2), and (3), respectively. In the case of the randomized *p*-value, an independent uniform variable, *X_i_*, is generated each time. ${\tilde{P}}_{0}$ denotes the implied joint probability measure of the statistics under ${\tilde{H}}_{0}$. The focus of this section is on testing the joint null hypothesis ${\tilde{H}}_{0}$. Probability bounds that follow often have the form ${\tilde{P}}_{0}\left\{f\left(Q_{1},\ldots ,Q_{n}\right)\geq t\right\}\leq b_{n}\left(t\right)$. If the observed mid-*p*-values are *q*_1_, …, *q_n_* and level of the test is α (e.g., 5%), then a procedure that rejects when *b_n_*{*f*(*q*_1_, …, *q_n_*)} ⩽ α is conservative: the probability of rejecting the null hypothesis ${\tilde{H}}_{0}$, if it holds, does not exceed α.

### 4.1. Sums of Mid-*p*-Values

An early advocate of mid-*p*-values, Barnard (1989, 1990) proposed to combine test results from different contingency tables by taking the sum of standardized mid-*p*-values. His exposition relies on some approximations. Our results make exact inference possible.

We begin with a bound on the sum of independent mid-*p*-values. This bound bears an interesting resemblance to Hoeff-ding’s inequality (Hoeffding 1963). It will later be extended to be relevant to Barnard’s analysis.

Theorem 1. Let *X*_1_, …, *X_n_* denote *n* independent sub-uniform random variables with mean ${\bar{X}}_{n}=n^{-1}\sum_{i=1}^{n}X_{i}$. Then, for 0 ⩽ *t* ⩽ 1/2,

(11) $$P\left(1/2-{\bar{X}}_{n}\geq t\right)\leq \min_{h\geq 0}{\left\{2e^{-ht}\sinh\left(h/2\right)/h\right\}}^{n},$$

(12) $$\leq \exp\left(-12nt^{2}\right){\left\{\sinh(6t)/(6t)\right\}}^{n},$$

(13) $$\leq \exp(-6nt^{2}).$$

A subuniform random variable has expectation 1/2 and is bounded between 0 and 1. Hoeffding’s inequality would therefore give us $P\left(1/2-{\bar{X}}_{n}\geq t\right)\leq \exp\left(-2nt^{2}\right)$ for 0 ⩽ *t* ⩽ 1/2, the cubic root. Our improvement is substantial, for example, suppose we observe an average of 0.4 from *n* = 100 mid-*p*-values. This is very significant: ${\tilde{P}}_{0}\left(1/2-{\bar{Q}}_{n}\geq 0.1\right)\leq 0.0025$ using (13). However, we would only find ${\tilde{P}}_{0}\left(1/2-{\bar{Q}}_{n}\geq 0.1\right)\leq 0.14$ using Hoeffding’s inequality.

Instead of summing the mid-*p*-values directly, Barnard (1990) actually considered sums of the standardized statistics

$$D_{i}=\left(1/2-Q_{i}\right)/\sigma_{i},$$

where σ_*i*_ is the standard deviation of *Q_i_* under ${\tilde{H}}_{0}$. The upper tail probability of the sum is then estimated by Gaussian approximation. In the purely discrete case, Barnard shows that σ_*i*_ = {(1 − *s_i_*)/12}^1/2^ where

$$s_{i}=\sum_{t\in S_{i}}{\left\{P_{0}^{\left(i\right)}\left(T_{i}=t\right)\right\}}^{3},$$

and *S_i_* is the (countable) support of *Q_i_*. Instead of appealing to the Gaussian approximation, the convex order allows us to find an exact bound.

Lemma 1. Let *X*_1_, …, *X_n_* denote *n* independent subuniform random variables with standard deviations σ_1_, …, σ_*n*_, respectively, and let

$${\bar{Y}}_{n}=\frac{1}{n}\sum_{i=1}^{n}\left(1/2-X_{i}\right)/\sigma_{i}.$$

Then, for *t* ⩾ 0,

(14) P(Y‾n≥t)≤minh≥0∏i=1nexp[-h{t+1/(2σi)}]×eh/σi-1sh/σi+h212-124σi2∏i=1n,

(15) $$\leq \exp\{-6n{\left(\bar{\sigma }t\right)}^{2}\},$$

where $\bar{\sigma }={\left(\prod \sigma_{i}\right)}^{1/n}$ is the geometric mean of the standard deviations.

In practice, the bound (14), which is an important improvement over (15), can be found numerically by minimizing over *h*. Of course, even if the optimum cannot be determined exactly the obtained bound still holds, because the tail area is simply over-estimated.

To illustrate how the bound (14) performs in practice, we now revisit Barnard’s example (Barnard 1990, p. 606). The first experiment he considers yields *Q*_1_ = 1/7, *s*_1_ = 9002/42^3^, *D*_1_ = 1.32. The second yields *Q*_2_ = 1/9, *s*_2_ = 141/729, *D*_2_ = 1.5. Since the sum divided by $\sqrt{2}$ is almost two, that is, two standard deviations away, he finds “serious evidence” against the null hypothesis. Lemma 1 gives ${\tilde{P}}_{0}\left(D_{1}+D_{2}\geq 1.32+1.5\right)\leq 0.12$, providing some evidence in favor of the alternative, but not significant at, say, the 5% level. On the other hand, evidence would start to become compelling if we were to observe the second result again, *Q*_3_ = 1/9, *s*_3_ = 141/729, *D*_3_ = 1.5; Lemma 1 then finds ${\tilde{P}}_{0}\left(D_{1}+D_{2}+D_{3}\geq 1.32+1.5+1.5\right)\leq 0.036$.

### 4.2. Products of Mid-*p*-Values (Fisher’s Method)

Fisher’s method (Fisher 1934) is the most popular way of combining *p*-values. As is well-known, under ${\tilde{H}}_{0}$, the statistic − 2∑^*n*^_*i* = 1_log (*P_i_*) has a chi-square distribution with 2*n* degrees of freedom if *P_i_* are absolutely continuous. Therefore, the *p*-value of the combined test is *P*† = *S*_2*n*_{ − 2∑^*n*^_*i* = 1_log (*P_i_*)}, where *S_k_* is the survival function of a chi-square distribution with *k* degrees of freedom. This results in an exact procedure when *P_i_* are absolutely continuous, and a conservative one otherwise, that is, *P*† ⩾ _*st*_*U* under ${\tilde{H}}_{0}$.

**Figure 2.**
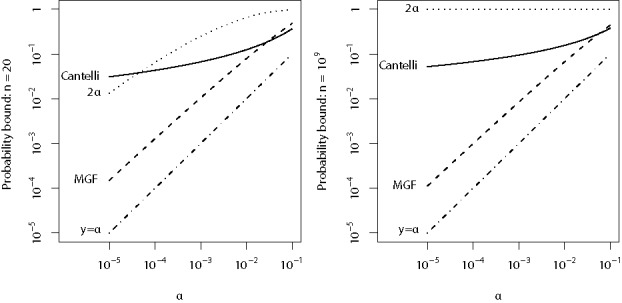
Comparison of the probability bounds given by Theorem 2 for Fisher’s method using mid-*p*-values. Theorem 2 gives explicit formulas for 2α, Cantelli and MGF, in that order. Both axes are on the logarithmic scale.

Our next result allows us to use the mid-*p*-values *Q*_1_, …, *Q_n_* in place of *P*_1_, …, *P_n_* while retaining a conservative procedure. We were able to derive three probability bounds. None beats the other two uniformly for all *n* and all significance levels (see Figure 2), but the last is often the winner, hence the simpler statement of Section 2.

Theorem 2. Let *X*_1_, …, *X_n_* be a sequence of independent sub-uniform random variables. Then for *x* ⩾ 2*n*,

$$\begin{array}{ccc} & & P\left(-2\sum_{i=1}^{n}\log\left(X_{i}\right)\geq x\right)\\ & & \;\leq \min\left[S_{2m}\left(x-2n\log2\right),n/\left[n+{\left\{\left(x-2n\right)/2\right\}}^{2}\right],\right.\\ & & \;\left.\exp\{n-x/2-n\log(2n/x)\}\right]=u_{n}\left(x\right). \end{array}$$

The first uses P(X_i_ ⩽ α) ⩽ 2α for α ⩾ 0, obvious in the case of a mid-*p*-value, but actually true of any subuniform random variable (Meng 1994). The second uses bounds on the mean and variance of − log (*X_i_*) (given in Lemma 2, in the Appendix) and then applies the Chebyshev-Cantelli inequality. The third is based on a bound on the moment generating function of − log (*X_i_*). Derivation details are in the Appendix.

For a given *n* and α ∈ (0, 1], let *t*_α, *n*_ denote the critical value of Fisher’s statistic, that is, *t*_α, *n*_ satisfies *S*_2*n*_(*t*_α, *n*_) = α. Figure 2 presents the behavior of the different bounds for different *n* (20 on the left and 1 billion on the right) and α. The curves show the bound given by each formula at different α (which can be interpreted as “canonical levels”), that is, inputting *x* = *t*_α, *n*_ in Theorem 2, as α ranges from 10^− 5^ to 0.1. For low α, the bound based on the moment generating function, marked MGF, is by far superior.

Let *Q*† = *u_n_*{ − 2∑^*n*^_*i* = 1_log (*Q_i_*)}. Then *Q*† is again conservative, that is, *Q*† ⩾ _*st*_*U* under ${\tilde{H}}_{0}$. Both *P*† and *Q*† are valid *p*-values. Clearly, if the underlying *p*-values are continuous, then the standard *P*† is superior (in fact, deterministically smaller). However, *Q*† seems to be substantially more powerful in a wide range of discrete cases. This is demonstrated by simulation in Section 4.3.

Finally, we find this interesting asymptotic result.

Theorem 3 (Fisher’s method is asymptotically conservative). Let *X*_1_, *X*_2_, … denote independent and identically distributed sub-uniform random variables. For any α ∈ (0, 1], there exists N∈N such that

$$P\left(-2\sum_{i=1}^{n}\log\left(X_{i}\right)\geq t_{\alpha ,n}\right)\leq \alpha ,$$

for any *n* ⩾ *N*.

Hence, we can dispense with any correction entirely if *n* is large enough and the *Q_i_* are identically distributed. A formal proof is given in the Appendix. Since $E\left\{-\log\left(X_{i}\right)\right\}\leq E\left\{-\log\left(U\right)\right\}$, from the definition of the convex order, a direct application of the law of large numbers gets us most of way, except for the possibility $E\left\{-\log\left(X_{i}\right)\right\}=E\left\{-\log\left(U\right)\right\}$. In fact, this exception is no problem because, perhaps surprisingly, it implies that the *X_i_* are uniform, using Shaked and Shanthikumar (2007, Theorem 3.A.43).

### 4.3. Simulations

**Figure 3.**
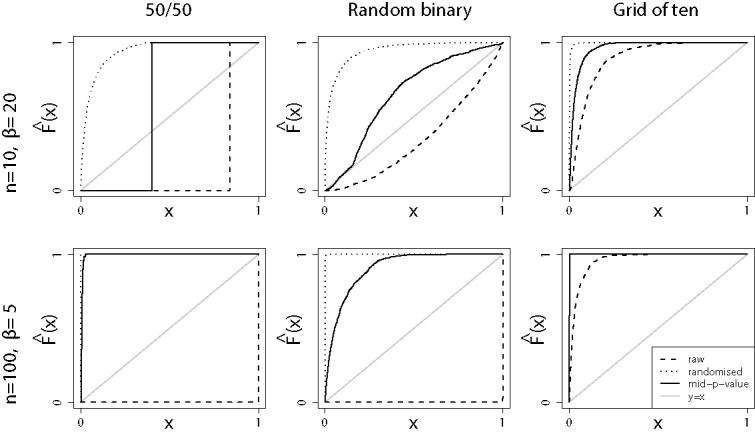
Fisher’s method with discrete *p*-values. Empirical distribution functions of Fisher’s combined *p*-value under different conditions. 50/50: Each *p*-value is equal to 1/2 or 1 (with probability 1/2 each under ${\tilde{H}}_{0}$). Random binary: Each *p*-value is equal to *p* or 1 (with probability *p* and 1 − *p*, respectively, under ${\tilde{H}}_{0}$). *p* is drawn uniformly on [0, 1] (independently of whether ${\tilde{H}}_{0}$ or ${\tilde{H}}_{1}$ holds). Grid of 10: Each *p*-value is drawn from 1/10, 2/10…, 1 (with probability 1/10 each under ${\tilde{H}}_{0}$). *n* = 10, β = 20: 10 *p*-values from a left-censored Beta(1, 20) distribution. *n* = 100, β = 5: 100 *p*-values from a left-censored Beta(1, 5) distribution. Dotted line: Randomized *p*-values. Solid line: Mid-*p*-value. Dashed line: Standard *p*-values. Further details in the main text.

To illustrate the potential improvement of employing Fisher’s method with mid-*p*-values, using the bound (7), over the traditional approach of using ordinary *p*-values and the chi-square tail, we considered *p*-values from three types of support. In the first column of Figure 3, each *p*-value *P_i_* can only take one of two values, 1/2 and 1. We therefore have *Q_i_* = 0.25 if *P_i_* = 1/2 and *Q_i_* = 0.75 if *P_i_* = 1. Under the null hypothesis, $P_{0}^{\left(i\right)}\left(P_{i}=1/2\right)=P_{0}^{\left(i\right)}\left(P_{i}=1\right)=1/2$. In the second column, each *p*-value *P_i_* is supported on the pair {*p_i_*, 1}, where *p_i_* were drawn uniformly on the unit interval but are subsequently treated as fixed known values. We have *Q_i_* = *p_i_*/2 if *P_i_* = *p_i_* and *Q_i_* = (1 + *p_i_*)/2 otherwise. Under the null hypothesis, we have $P_{0}^{\left(i\right)}\left(P_{i}=p_{i}\right)=1-P_{0}^{\left(i\right)}\left(P_{i}=1\right)=p_{i}$, for each *i*. Finally, in the third column each *p*-value *P_i_* takes one of 10 values, 1/10, 2/10, …, 1, and therefore *Q_i_* = *P_i_* − 1/20. Under the null hypothesis, P^(i)^_0_(P_i_ = j/10) = 1/10, for *j* = 1, …, 10. The rows represent two different alternatives and sample sizes. In both cases, the *P_i_* are generated by left-censoring a sequence of independent and identically distributed Beta variables, *B*_1_, …, *B_n_*, that is, *P_i_* is the smallest supported value larger than *B_i_*. In the first scenario, the dataset is small (*n* = 10), but the signal is strong (a Beta distribution with parameters 1 and 20). In the second, the dataset is larger (*n* = 100) but the signal is made weaker accordingly (a Beta distribution with parameters 1 and 5). Comparing just the solid and dashed lines first, we see that *Q*† always outperforms *P*† substantially, and sometimes overwhelmingly. In the bottom-left corner, for example, we have a situation where, at a false positive rate set to 5% say, the test *Q*† would detect the effect with probability close to one whereas with *P*† the probability would be close to zero.

As a final possibility, consider *R*† = *S*_2*n*_{ − 2∑^*n*^_*i* = 1_log (*R_i_*)}. A disappointment is that this randomized version, the dotted line in Figure 3, tends to outperform even the mid-*p*-values, and by a substantial margin. On the other hand, as pointed out in the introduction, the randomized *p*-value has some important philosophical disadvantages, and did not perform better in our real data example.

## 5. Conclusion

The convex order provides a formal platform for the treatment and interpretation of mid-*p*-values. This article used mathematical results from this literature to combine mid-*p*-values, which are not conservative individually, into an overall significance level that is conservative. As shown in real data and simulations, the gains in power can be substantial.

Whereas the focus of this article was on meta-analysis, another canonical problem is multiple testing, where the task is to subselect from or adjust a set of *p*-values, for example, subject to a maximum false discovery rate (Benjamini and Hochberg 1995). The case of discrete data has been analyzed in a number of articles, including Kulinskaya and Lewin (2009); Habiger and Pena (2011); Liang (2016); Habiger (2015). A promising (but ostensibly harder) avenue of research would be to investigate the use of the convex order in this problem.

## Appendix: Mathematical Details and Proofs

### A1. Section 3: Mathematical Details

First, we calculate the empirical rates

$$r_{j}=\frac{\text{number}\;\text{of}\;\text{new}\;\text{connections}\;\text{to}\;j\;\text{over}\;\left[0,58\right]}{\text{(number}\;\text{of}\;\text{nodes}\;\text{-}\;\text{1)}\times 58},$$

for *j* = 1, …, *m*, and then set $\alpha ={\left\{\text{mean}\left(r_{j}\right)\right\}}^{2}/\text{var}\left(r_{j}\right)$, $\beta =\text{mean}\left(r_{j}\right)/\text{var}\left(r_{j}\right)$, so that a Gamma distribution with parameters α and β has mean and variance equal to the empirical mean and variance of *r_j_*, respectively. This distribution is used as the prior for each rate λ_*j*_. After a day of observation, the posterior distribution for λ_*j*_ is also Gamma, with parameters

$$\alpha_{j}=\alpha +\#\left\{\text{new}\;\text{connections}\;\text{to}\;j\;\text{over}\;\left[0,1\right)\right\};\;\beta_{j}=\beta +\sum \tau_{ij},$$

where

$$\tau_{ij}=\left\{\begin{array}{cc} 1 & \text{if}\;\text{no}\;\text{connection}\;\text{occurs}\;\text{from}\;i\;\text{to}\;j\;\text{in}\;\left[0,1\right)\text{,}\\ \tau & \text{if}\;\text{a}\;\text{new}\;\text{connection}\;\text{from}\;i\;\text{to}\;j\;\text{occurs}\;\text{at}\;\text{time}\;\tau <1. \end{array}\right.$$

For each *i*, restrict *j* to the indices of nodes that did not receive a connection from *i* over [0, 1). Conditional on the observation period [0, 1), each statistic *T_ij_* has a probability measure, denoted P^(ij)^_0_, with a point mass at 57 and an absolutely continuous distribution over [0, 57) given by

$$\begin{array}{ccc} P_{0}^{\left(ij\right)}\left(T_{ij}\leq t\right) & = & 1-\int_{0}^{\infty }e^{-\lambda_{j}t}\cdot \frac{\beta_{j}^{\alpha_{j}}}{\Gamma (\alpha_{j})}\lambda_{j}^{\alpha_{j}-1}e^{-\beta_{j}\lambda_{j}}d\lambda_{j}\\ & = & 1-{\left(1+\frac{t}{\beta_{j}}\right)}^{-\alpha_{j}},\;\text{for}\;t\in [0,57),\\ P_{0}^{\left(ij\right)}\left(T_{ij}=57\right) & = & {\left(1+57/\beta_{j}\right)}^{-\alpha_{j}}. \end{array}$$

Since low values of *T_ij_* are of interest, the *p*-value and mid-*p*-value associated with *T_ij_* are computed from lower-tailed versions of (1) and (2) by substituting P^(ij)^_0_ in for P_0_, giving

$$P_{ij}=\left\{\begin{array}{cc} 1, & T_{ij}=57,\\ 1-{\left(1-T_{ij}/\beta_{j}\right)}^{-\alpha_{j}}, & T_{ij}<57, \end{array}\right.$$

and

$$Q_{ij}=\left\{\begin{array}{cc} 1/2+\{1-{\left(1-57/\beta_{j}\right)}^{-\alpha_{j}}\}/2, & T_{ij}=57,\\ 1-{\left(1-T_{ij}/\beta_{j}\right)}^{-\alpha_{j}}, & T_{ij}<57, \end{array}\right.$$

respectively.

### Proofs

Proof of Theorem 1. Since 1 − *X* is subuniform if and only if *X* is sub-uniform, it is sufficient to prove the bounds in (11), (12), and (13) hold for $P({\bar{X}}_{n}-1/2\geq t)$. Since exp (*xh*) is a convex function in *x* for any *h*, the convex order gives us $E\left\{\exp\left(hX_{i}\right)\right\}\leq E\left\{\exp\left(hU\right)\right\}=\left(e^{h}-1\right)/h$. Therefore, for any *h* ⩾ 0,

$$\begin{array}{ccc} P\left({\bar{X}}_{n}-1/2\geq t\right) & = & P\left[\exp\left(\sum_{i=1}^{n}hX_{i}\right)\geq \exp\left\{nh\left(t+1/2\right)\right\}\right],\\ & \leq & \exp\left\{-nh\left(t+1/2\right)\right\}E\left\{\exp\left(\sum_{i=1}^{n}hX_{i}\right)\right\},\\ & \leq & \exp\left\{-nh\left(t+1/2\right)\right\}{\left\{\left(e^{h}-1\right)/h\right\}}^{n}\\ & = & {\left\{2e^{-ht}\sinh\left(h/2\right)/h\right\}}^{n}, \end{array}$$

where the second line follows from Markov’s inequality. The choice *h* = 12*t* (motivated by an analysis of the Taylor expansion in *h* at 0) leads to

$$\begin{array}{ccc} P\left({\bar{X}}_{n}-1/2\geq t\right) & \leq & \exp\left(-12nt^{2}\right){\left\{\sinh(6t)/(6t)\right\}}^{n}\\ & \leq & \exp\left(-6nt^{2}\right){\left\{e^{-6t}\sinh\left(6t\right)/\left(6t\right)\right\}}^{n}\leq \exp\left(-6nt^{2}\right), \end{array}$$

using the fact that *e*^− *x*^sinh (*x*)/*x* = (1 − *e*^− 2*x*^)/(2*x*) is one at *x* = 0 (using l’Hospital’s rule) and decreasing.

Proof of Lemma 1. Again, we will prove the bound holds for *W_n_* = *n*^− 1^∑(*X_i_* − 1/2)/σ_*i*_, so that the theorem holds by symmetry. For any *h* ⩾ 0,

$$\begin{array}{ccc} E\{\exp\left(hX_{i}/\sigma_{i}\right)\} & = & 1+E\left(hX_{i}/\sigma_{i}\right)+E\left\{{\left(hX_{i}/\sigma_{i}\right)}^{2}\right\}/2+\cdots \\ & = & 1+E\left(hU/\sigma_{i}\right)+h^{2}\left(\frac{1}{2}+\frac{1}{8\sigma_{i}^{2}}\right)+\cdots \\ & \leq & E\left\{\exp\left(hU/\sigma_{i}\right)\right\}+h^{2}\left(\frac{1}{2}+\frac{1}{8\sigma_{i}^{2}}-\frac{1}{6\sigma_{i}^{2}}\right), \end{array}$$

because $E\left\{{\left(hX_{i}/\sigma_{i}\right)}^{n}\right\}\leq E\left\{{\left(hU/\sigma_{i}\right)}^{n}\right\}$ for *n* ⩾ 3, by the convex order, and E{(U/σ_i_)^2^}/2 = 1/(6σ^2^_i_). Therefore,

$$\begin{array}{ccc} P(W_{n}\geq t) & = & P\left[\exp\left\{\sum_{i=1}^{n}h\left(X_{i}-1/2\right)/\sigma_{i}\right\}\geq e^{hnt}\right],\\ & \leq & e^{-hnt}E\left[\exp\left\{\sum_{i=1}^{n}h\left(X_{i}-1/2\right)/\sigma_{i}\right\}\right],\\ & = & \prod_{i=1}^{n}\exp\left[-h\left\{t+1/\left(2\sigma_{i}\right)\right\}\right]\left\{\frac{e^{h/\sigma_{i}}-1}{h/\sigma_{i}}+h^{2}\left(\frac{1}{2}-\frac{1}{24\sigma_{i}^{2}}\right)\right\}, \end{array}$$

proving that (14) holds. Next, since σ^2^_*i*_ ⩽ 1/12,

$$\begin{array}{ccc} P(W_{n}\geq t) & \leq & \prod_{i=1}^{n}\exp\left[-h\left\{t+1/\left(2\sigma_{i}\right)\right\}\right]\left(\frac{e^{h/\sigma_{i}}-1}{h/\sigma_{i}}\right)\\ & = & {\left(2e^{-ht}{\left[\prod_{i=1}^{n}\sin h\left\{h/\left(2\sigma_{i}\right)\right\}\right]}^{1/n}/\left(h/\bar{\sigma }\right)\right)}^{n}\\ & \leq & {\left\{2e^{-ht}\sin h\left(h/\left(2\bar{\sigma }\right)\right)/\left(h/\bar{\sigma }\right)\right\}}^{n}, \end{array}$$

using the fact that the function sinh  is geometrically convex on [0, ∞) (Niculescu 2000). We proceed as in the proof of Theorem 1, choosing $h=12\bar{\sigma }t$.

The proofs of Theorems 2 and 3 both need the following result.

Lemma 2. Let *X* be a sub-uniform random variable. Then either (i) *X* is uniform on [0, 1] or (ii)

E{-log(X)}<E{-log(U)}=1;var{-log(X)}<var{-log(U)}=1,

where *U* is a uniform random variable on [0, 1].

Proof. Shaked and Shanthikumar (2007, Theorem 3.A.43) provide the following theorem. If *X* ⩽ _*cx*_*Y* and for some strictly convex function *h* we have E{h(X)}=E{h(Y)} then *X* is distributed as *Y*. The function − log (*x*) is strictly convex, therefore either *X* is uniform or E{-log(X)}<E{-log(U)}. If the latter is true, then

$$\begin{array}{ccc} \mathrm{var}\{-\log(X)\} & = & E{\left[-\log\left(X\right)-E\left\{-\log\left(X\right)\right\}\right]}^{2}\\ & < & E{\left[-\log\left(X\right)-E\left\{-\log\left(U\right)\right\}\right]}^{2}\\ & \leq & E{\left\{\log\left(U\right)+1\right\}}^{2}\\ & = & \mathrm{var}\{-\log(U)\}. \end{array}$$

In the second line, the fact that the expected squared distance from the mean is smaller than from any other point is used, and in the fourth we used the fact that (log (*x*) + 1)^2^ is convex.

Proof of Theorem 2. Let *G_n_* = −2∑log (*X_i_*). Now, *U_i_*/2 ⩽ _*st*_*X_i_*, for *i* = 1, …, *n*, where *U*_1_, …, *U_n_* are independent uniform random variables on [0, 1]. This implies − log (*X_i_*) ⩽ _*st*_ − log (*U_i_*/2). Because the usual stochastic order is closed under convolution (Shaked and Shanthikumar 2007, Theorem 1.A.3), we have *G_n_* ⩽ _*st*_ − 2∑log (*U_i_*) + 2*n*log 2. The sum − 2∑log (*U_i_*) has a chi-square distribution with 2*n* degrees of freedom, proving the first bound. Lemma 2 implies E(G_n_) ⩽ 2n and $\mathrm{var}(G_{n})\leq 4n$. Therefore, using Cantelli’s inequality,

$$\begin{array}{ccc} P[G_{n}\geq x] & \leq & \mathrm{var}\left(G_{n}\right)/\left[\mathrm{var}\left(G_{n}\right)+{\left\{x-E\left(G_{n}\right)\right\}}^{2}\right]\\ & \leq & \mathrm{var}\left(G_{n}\right)/\left[\mathrm{var}\left(G_{n}\right)+{\left\{x-2n\right\}}^{2}\right]\\ & \leq & n/\left[n+{\left\{\left(x-2n\right)/2\right\}}^{2}\right], \end{array}$$

for *x* ⩾ 2*n*. This proves the second bound. Finally, the moment generating function of *G_n_* is $E\left\{\exp\left(tG_{n}\right)\right\}=\prod E\left(X_{i}^{-2t}\right)$ for *t* ⩾ 0. For *t* ∈ [0, 1/2) each $E\left(X_{i}^{-2t}\right)\leq E\left(U^{-2t}\right)={\left(1-2t\right)}^{-1}$ since *x*^− 2*t*^ is a convex function in *x* for *x* ∈ [0, 1]. Using Markov’s inequality,

$$\begin{array}{ccc} P(G_{n}\geq x) & = & P\{\exp\left(tG_{n}\right)\geq \exp\left(tx\right)\}\\ & \leq & \exp\left(-tx\right)E\{\exp\left(tG_{n}\right)\}\\ & \leq & \exp(-tx-n\log(1-2t)), \end{array}$$

for *t* ∈ [0, 1/2). The minimum of this function is at *t* = 1/2 − *n*/*x*, giving the third bound.

Proof of Theorem 3. Let *V_i_* = −2log (*X_i_*), μ_*V*_ = E(V_i_), *W_i_* = −2log (*U_i_*), where *U*_1_, …, *U_n_* are independent uniform random variables on [0, 1], and μ_*W*_ = E(W_i_). If μ_*V*_ = μ_*W*_ then by Lemma 2 the *X_i_* are uniform on [0, 1] and we are done. The statement is also true if α = 1. Therefore assume μ_*V*_ < μ_*W*_, α ∈ (0, 1) and let *t* ∈ (μ_*V*_, μ_*W*_). By the weak law of large numbers there exists an $N^{'}\in N$ such that, for *n* ⩾ *N*′,

$$P\left(\sum_{i=1}^{n}W_{i}\geq nt\right)\geq \alpha ,$$

so that *t*_α, *n*_ ⩾ *nt*. Therefore, for *n* ⩾ *N*′,

$$\begin{array}{c} P\left(\sum_{i=1}^{n}V_{i}\geq t_{\alpha ,n}\right)\leq P\left(\sum_{i=1}^{n}V_{i}\geq nt\right). \end{array}$$

Again by the law of large numbers, the right-hand side tends to zero. Hence, there exists an *N* ⩾ *N*′ such that it is bounded by α for *n* ⩾ *N*.
